# In Vitro Assessment of Essential Oils for Their Methane Mitigation Potential and Impact on Rumen Fermentation in Cattle

**DOI:** 10.3390/ani16030373

**Published:** 2026-01-24

**Authors:** Memoona Nasir, Rokia Temmar, Abdelhacib Kihal, José Luis Repetto, Cecilia Cajarville, Gwenael Forgeard, Jihane Guihard, María Rodríguez-Prado, Susana M. Martín-Orúe, José Francisco Pérez, Sergio Calsamiglia

**Affiliations:** 1 Animal Nutrition and Welfare Service (SNiBA), Department of Animal and Food Science, Universitat Autònoma de Barcelona (UAB), 08193 Bellaterra, Spain; temmarrokia@gmail.com (R.T.); abdelhacib.khl@gmail.com (A.K.); joselorepetto@gmail.com (J.L.R.); ccajarville@gmail.com (C.C.); maria.rodriguez.prado@uab.cat (M.R.-P.); susana.martin@uab.cat (S.M.M.-O.); josefrancisco.perez@uab.cat (J.F.P.);; 2 TECHNA France Nutrition, BP 10, 44220 Couëron, France; gwenael.forgeard@groupe-techna.com (G.F.); jihane.guihard@groupe-techna.com (J.G.)

**Keywords:** essential oils, feed additives, garlic oil, in vitro, methane mitigation, microbial fermentation, terpenoid

## Abstract

Strategies to suppress methanogenesis must preserve the functional integrity of the rumen microbial ecosystem. Essential oils (EOs) have emerged as promising modulators of rumen microbial function, though their responses vary widely with chemical structure and inclusion level. This study evaluated the efficacy of selected EOs using detailed in vitro fermentation assays. Nine EOs—cinnamon, lavender, garlic (GAR), lemongrass (LEG), peppermint (PPM), eucalyptus, coriander, oregano, and ginger (GIN)—were evaluated for their effects on rumen fermentation and methane (CH_4_) production using a 24 h in vitro batch culture system. Eight EOs were tested at two doses (Low and High) specific to each EO, while GIN was evaluated at a single dose. All treatments were incubated in a rumen fluid–buffer mix (1:1 for fermentation parameters and 1:4 for gas and CH_4_ measurements) with a 55:45 forage-to-concentrate substrate (pH 6.9). Overall treatment effects were significant for all measured fermentation parameters (*p* < 0.01). Most treatments reduced total gas production, CH_4_ emissions, and CH_4_/total gas ratios compared with the control (*p* < 0.05), although several responses were dose-dependent or directly divergent. Essential oils showed clear, composition-dependent responses: non-terpenoid EOs produced the strongest but also the most variable antimethanogenic effects, with GAR, particularly at the lower dose, consistently achieving the greatest CH_4_ inhibition while maintaining a favorable fermentation pattern. Conversely, terpenoid-based EOs induced moderate, dose-responsive CH_4_ reductions with minimal effects on overall fermentation. At the higher dose, PPM suppressed CH_4_ without altering major volatile fatty acid (VFA) patterns aside from increases in valerate and branched-chain VFA, whereas LEG reduced CH_4_ only when accompanied by marked fermentation depression. Monensin validated its role as an effective positive control. Overall, GAR, characterized by sulfur-based bioactives, emerged as the most effective candidate for CH_4_ mitigation under the tested in vitro conditions, highlighting the importance of chemical composition and inclusion level in determining efficacy and reinforcing the need for in vivo validation.

## Simple Summary

Essential oils are a diverse group of plant-derived compounds with growing potential to improve rumen fermentation while addressing the pressing challenge of methane emissions. These natural bioactives, defined by distinct chemical structures and mechanisms, can modulate rumen microbial activity in complex, dose-dependent ways. In this study, several terpenoid and non-terpenoid essential oils were evaluated under in vitro rumen conditions to determine how oil composition and inclusion level influence fermentation and methanogenesis. The results showed clear composition-linked responses: terpenoid essential oils produced moderate, dose-dependent reductions in methane with minimal impact on fermentation, whereas non-terpenoid oils exerted stronger but more variable effects. Among them, garlic oil, characterized by its sulfur-based constituents, achieved the greatest methane inhibition, while maintaining a generally favorable fermentation pattern, despite a moderate decrease in overall fermentation intensity. Overall, the findings indicate that essential oils can serve as promising natural tools for enteric methane mitigation, with garlic oil showing the most consistent effects under the tested conditions, whereas other oils displayed benefits only within narrower application ranges. As efficacy is inherently governed by both chemical composition and inclusion level, these results highlight the need for precise formulation to balance methanogenic inhibition with rumen fermentative stability.

## 1. Introduction

The rumen is a specialized anaerobic ecosystem where methanogenic archaea convert hydrogen (H_2_) and CO_2_ into methane (CH_4_) to maintain redox balance [1]. Although essential for fermentation, this process diverts 2–12% of dietary energy [2] and produces CH_4_, a greenhouse gas 28 times more potent than CO_2_ [3]. Enteric fermentation is the largest source of agricultural CH_4_ and accounts for approximately 30% of global anthropogenic CH_4_ emissions [4,5], making rumen methanogenesis a critical target for mitigation. Because CH_4_ has a short atmospheric lifetime, reducing enteric emissions offers rapid climate benefits [6] and aligns with international initiatives such as the Global CH_4_ Pledge [7]. As methanogenesis is strongly influenced by diet [8], dietary strategies have become central to CH_4_ mitigation efforts [9]. Feed additives play an important role in this context, enhancing ruminant productivity by improving nutrient utilization and fermentation efficiency [10]. Following the EU ban on antibiotic growth promoters [11], and reflecting a broader global shift toward natural feed additives driven by sustainability and CH_4_ mitigation goals [9,12], attention has increasingly focused on natural bioactive compounds that enhance rumen fermentation while limiting CH_4_ output [13].

Essential oils (EOs), volatile, plant-derived compounds, with recognized antimicrobial and antioxidant properties [14,15], have gained increasing attention as natural rumen-modifying agents. Their biological activity in the rumen arises from diverse biochemical mechanisms [16] that are strongly influenced by their dominant chemical constituents [17]. Extensive research has demonstrated that under certain conditions, EOs can reduce CH_4_ emissions and effectively alter the fatty acid profiles in ruminant-derived products [18,19]. For instance, in vitro studies with garlic oil have shown notable reductions in CH_4_ production and methanogen abundance, alongside a decreased acetate-to-propionate ratio (A:P) while maintaining volatile fatty acid (VFA) production [20,21]. However, the literature remains highly inconsistent. Some studies report adverse effects on rumen fermentation parameters [22,23] while others show no measurable impact [24]. Considerable uncertainty also remains regarding optimal dosing and the comparative performance of chemically distinct EOs [25]. These inconsistencies highlight the need to evaluate a broader spectrum of EOs representing diverse mechanisms of action.

A key challenge in EO research is their pronounced dose-dependence. Their efficacy is tightly linked to concentration [26], and the margin between desirable and adverse effects is often narrow [27]. At low inclusion levels, EOs frequently exert minimal influence on fermentation [28], whereas higher doses can markedly increase antimicrobial pressure, sometimes resulting in broad suppression of the rumen microbiota [22]. Importantly, the inclusion levels required to achieve meaningful reductions in CH_4_ emissions may approach or exceed those at which negative fermentation effects begin to appear [29]. These complex and sometimes contradictory dose–response patterns highlight the need for precise supplementation strategies and a clearer mechanistic understanding of how individual EOs interact with rumen microbial communities to influence CH_4_ production and nutrient utilization. Despite extensive work on individual EOs, relatively few studies have systematically compared multiple chemically distinct EOs under standardized in vitro conditions that allow direct comparison across additives. Even fewer have evaluated their dose-dependent effects using complementary fermentation and gas-production assays. As a result, most existing studies rely on single-dose screening or assess either VFA profiles or CH_4_ output in isolation, limiting mechanistic interpretation. The present study addresses these gaps by systematically evaluating structurally diverse EOs at two inclusion levels within a unified experimental framework that integrates fermentation profiles with gaseous outputs. This approach enables direct comparison of additive selectivity, dose-responsiveness, and the balance between CH_4_ reduction and fermentative preservation, providing mechanistic insight beyond typical single-outcome screening studies.

This study aimed to investigate the effects of selected EOs on in vitro rumen fermentation and CH_4_ production using a batch-culture system. It was hypothesized that supplementation with these oils would modulate rumen fermentation and CH_4_ emission, with the magnitude of response varying according to oil type and inclusion level. Dose-dependent effects were therefore anticipated for the EOs evaluated at more than one inclusion level.

## 2. Materials and Methods

### 2.1. Study Design and Ethical Approval

This study aimed to assess the impact of different treatments on microbial fermentation parameters and CH_4_ production, using a single experiment evaluated through two complementary in vitro approaches. Each approach was conducted across two independent incubation periods, using freshly collected rumen fluid to ensure biological replication. Approach 1 evaluated the influence of treatments on ammonia-N (NH_3_-N) concentration, and the concentration and molar distribution of VFAs using a modified procedure of Tilley and Terry [30]. Approach 2 subsequently assessed gas and CH_4_ production by applying a pressure transducer-based incubation technique [31]. Rumen fluid was collected in each experimental period from two mature Holstein dairy cows (approximately 650 kg body weight), fitted with permanent ruminal cannulae. The donor animals were non-pregnant, non-lactating, and were maintained under standardized husbandry conditions on a consistent maintenance ration composed of 60% alfalfa hay and 40% concentrate (DM basis). Experimental protocols received prior approval from the Animal Experimentation Ethics Committee of the Universitat Autònoma de Barcelona (UAB) and authorization from the Competent Authorities of the Generalitat de Catalunya (Ref: DGPAMN 10922). All procedures strictly adhered to the European Union regulations governing the ethical use and welfare of animals in research.

### 2.2. Experimental Treatments and Incubation Substrate

The experiment tested 10 additives, comprising nine plant EOs and monensin (MON), which served as a positive control to verify that the in vitro system responded appropriately to a well-established modulator of rumen fermentation and methanogenesis. Eight EOs treatments were assessed at two doses, whereas GIN and MON were administered at single doses. Each incubation set also included a negative control (CTR) without any additives and a blank (no diet or additive), which served as a correction factor. TECHNA France Nutrition (Couëron, France) provided all the EOs used in this study, while Monensin sodium salt (M5273) was sourced from Sigma-Aldrich (Sigma-Aldrich, St. Louis, MO, USA). The specific treatments and their respective inclusion concentrations, proposed by TECHNA and subsequently aligned with concentration ranges commonly reported in previous in vitro screening studies [32,33,34,35,36,37], are detailed in Table 1. The experimental treatments were coded by combining each compound additive with its corresponding dose (Low, High, or Single), for instance: CIN_Low; COR_High; GIN_SNGL, and so forth.

The inclusion levels for each essential oil were categorized as “Low” and “High” based on preliminary dose–response screening trials to identify effective concentration ranges. The Low doses corresponded to the minimum effective concentrations shown to elicit measurable yet selective changes in rumen fermentation without broad microbial suppression, whereas the High doses represented the upper functional limits identified in screenings to maximize response while maintaining microbial selectivity. This dual-dose approach enabled evaluation of concentration-dependent effects within a biologically relevant range, acknowledging that essential oil responses are compound-specific and dosage dependent.

Solutions of the different EO and MON were dissolved in ethanol (96% *v*/*v*) at the necessary concentration to achieve the final dose in the incubation bottle [38]. The same equivalent amount of ethanol was added to the CTR, and blank treatments to maintain consistency across treatments.

The diet used as a substrate was a 55:45 forage-to-concentrate ratio, chosen as a representative mixed ration that supports balanced rumen fermentation while providing a realistic baseline CH_4_ output for assessing treatment effects. The substrate was added in a proportion of 0.1 g for each 10 mL of inoculum media, and consisting of 55% grass hay, 24% ground grain corn, 20.1% soybean meal, and 0.9% trace mineralized mix. Each ingredient was ground to pass through a 1-mm screen sieve. The diet’s nutrient composition (% DM basis) was 16.3% CP (crude protein), 43.4% NDF (neutral detergent fiber), and 18.4% ADF (acid detergent fiber) and 85.7% OM (organic matter).

Chemical composition of the substrate was determined following standard analytical procedures. The diet sample was analyzed for dry matter (DM) using a forced-air oven at 103 °C for 24 h [39]. Total CP (N × 6.25) content was determined using the Kjeldahl method [39], while the NDF and ADF contents were determined sequentially by the filter bag procedure (Ankom Technology Corp., Macedon, NY, USA) with the inclusion of heat-stable α-amylase, sodium sulfite, and correction for insoluble ash content.

### 2.3. Rumen Fluid Collection

On the day of incubation, approximately 4 L of rumen fluid was collected at 08:00 h from a cannulated dairy cow following a 12 h fasting period, a standard procedure used to minimize diurnal variation and ensure consistency in the inoculum before morning feeding. The collected rumen fluid was immediately filtered through four layers of cheesecloth and transferred to the laboratory within 15 min in a pre-warmed, airtight thermo container. Once in the laboratory, the rumen fluid was homogenized and subsequently used to formulate the inoculum medium for the two in vitro incubation approaches.

### 2.4. Approach 1: Evaluating Rumen Microbial Fermentation

The inoculum was prepared by mixing rumen fluid with McDougall’s buffer [40] at a 1:1 (*v*/*v*) ratio. The pH was adjusted to 6.9 using HCl (37% *v*/*v*) or 6 N NaOH as appropriate. The inoculum medium was continuously homogenized using a magnetic stirrer and maintained at 39 °C in a thermo-regulated water bath agitated at 70 rpm, under a nitrogen-free O_2_ gas stream until inoculation to ensuring anaerobic conditions.

Incubations were conducted in duplicate as technical replicates within each incubation period, and the procedure was repeated in two independent periods to provide biological replication. Each incubation used 100 mL polypropylene tubes filled with 70 mL of incubation media, 0.7 g of diet, and 0.35 mL of either the treatment solution or ethanol. These proportions follow the established bottle-based in vitro fermentation framework of Goering and Van Soest [41], which employs ≈ 1:100 substrate-to-liquid ratios to maintain adequate buffering capacity, substrate availability, and headspace for microbial activity. After dispensing the inoculation media, the tubes were flushed with N-free O_2_ gas, fitted with gas-release rubber stoppers, placed in an agitated, temperature-controlled water bath at 39 °C, and incubated for 24 h. Samples were collected at 6 and 24 h, and frozen for later analysis of NH_3_-N and short-chain VFA. Samples for VFA analysis were pooled by treatment replicates within each period and stored frozen until analysis. pH was measured at the start and end of incubation using a portable digital pH meter (sensION+ Model PH31, HACH, Barcelona, Spain).

#### Chemical Analysis

For VFA analysis, within each incubation period, duplicate tubes for a given treatment were pooled to obtain sufficient volume for analysis and to reduce analytical variability following the approach described by Jouany [42]. Because pooling removes within-period variation, VFA measurements represent pooled technical replicates, and statistical inference relied on the two incubation periods as the biological replicates. Pooled samples for short-chain VFA analysis were then prepared following the method described by Jouany [42] with given modifications. In brief, 0.5 mL of a solution made up of a 0.2% (*wt*/*wt*) solution of mercuric chloride, 0.2% (*wt*/*wt*) of 4-methyl valeric acid as an internal standard, and 2% (*v*/*v*) orthophosphoric acid, was added to 2 mL of the sample and frozen. After being thawed, samples were centrifuged at 3000× *g* for 30 min, and the supernatant was analyzed via gas chromatography (Hewlett Packard, Palo Alto, CA, USA).

For NH_3_-N analysis, 1 mL of sample was mixed with 1 mL of 0.2 N HCl and stored at −20 °C. After thawing, the samples were centrifuged at 15,000× *g* for 20 min at 7 °C, and the resulting supernatant was analyzed colorimetrically following the procedure of Chaney and Marbach [43].

### 2.5. Approach 2: Evaluating Gas and CH_4_ Production

The procedure followed was similar to that previously described for assessing ruminal microbial fermentation, with the following modifications: (1) The inoculum medium was prepared by mixing rumen fluid with buffer solution [41] at a 1:4 (*v*/*v*) ratio to minimize excessive gas production and accumulation. The microminerals and resazurin omitted as recommended by Mould et al. [44]. (2) Incubations were carried out in quadruplicate using 60-mL serum bottles over two experimental periods. Each bottle contained 40 mL of incubation medium, 0.4 g of diet substrate, and 0.20 mL of either the treatment solution or ethanol. These volumes follow the sealed-bottle gas production technique of Theodorou et al. [31], which was developed as an adaptation of the bottle-based fermentation system originally established by Goering and Van Soest [41]. Two bottles were allocated for measuring total gas production, while the other two were used for CH_4_ determination. (3) Immediately after dispensing the inoculum medium, the bottles were flushed with nitrogen-free O_2_ gas, sealed with butyl rubber stoppers and aluminum caps, and incubated for 24 h at 39 °C in a water bath.

#### Measurement, Sampling, and Analysis

At the start of incubation and after each pressure reading, the bottles were gently agitated. Following each incubation interval, the headspace pressure was released just after incubation to attain pressure at point 0 and was subsequently recorded at 2, 4, 6, 8, 10, and 24 h in two of the four replicates. Pressure monitoring was carried out using a TP804 transducer connected to an HD-8804 gauge (DELTA OHM, Padova, Italy). After every measurement, accumulated gas was vented until the headspace returned to equilibrium (0.0 KPascal).

For CH_4_ determination, gas released from two replicates was collected using a needle attached to a BD Vacutainer^®^ holder (BD Vacutainer, Plymouth, UK) and injected into 12-mL glass vials sealed with Exetainer^®^ caps containing pierceable chlorobutyl rubber septa (Labco, Lampeter, UK). Samples were maintained at ambient temperature until analysis. CH_4_ levels were then measured by gas chromatography equipped with a thermal conductivity detector (GC–TCD), applying the ASTM D1945 standard procedure (Rev. 3146; ASTM International, West Conshohocken, PA, USA) [45], with a lower detection threshold of 10 ppmv.

Upon completion of the incubation period, the bottles were unsealed, and pH was measured using the previously specified pH meter.

### 2.6. Experimental Design, Data Calculations, and Statistical Analysis

The study was designed as a randomized block design, with two factors: treatments, administered at single or two doses, and two blocks (periods) with two replications in each block for each of the response variables. All data were subjected to analysis using the SAS software system (SAS version 9.4; SAS Institute Inc., Cary, NC, USA).

A linear regression of pressure by volume (Volume (mL) = 1.1936 (KPascal) + 0.0022, r^2^ = 1.00), based on measurements of 8 known volumes (ranging from 1 to 100 mL), each measured 5 times, was employed to obtain the volume of gas produced at each reading. The obtained volume was then adjusted with pressure values registered in the blanks, which were subtracted from the pressure readings of the treatments at each incubation time. The cumulative gas produced was calculated as the sum of each volume of gas generated at each incubation time and then corrected by the mass of the substrate incubated (expressed as mL of gas/g DM of the substrate).

The CH_4_ concentration was quantified as a fraction of the total gas produced. Concentrations were initially determined in parts per million (ppm), with a conversion factor of 10,000 ppm corresponding to 1% CH_4._ Cumulative CH_4_ production was calculated in parallel with cumulative gas production by summing the CH_4_ volume generated at each incubation interval. The resulting values were normalized to the amount of substrate incubated and expressed as mg CH_4_/g DM. Additionally, the CH_4_ ratio at 24 h was calculated as the volume of CH_4_ produced after 24 h (mL/g DM) divided by the total gas volume produced (GP) after 24 h (mL/g DM), and expressed as a percentage, according to:

CH_4_(mL/mL_total gas24h_) = [CH_4_ (mL/g DM)/GP_24_ (mL/g DM)] × 100

Data analysis was performed using the PROC MIXED procedure in SAS. The model included the fixed effects of treatment and block (period), with each incubation flask within treatments considered a random effect. For VFA and NH_3_-N analysis, the effect of sampling time (6 and 24 h post-incubation) was also included as a fixed-term effect. Treatment was defined as the combination of each additive dosed at low, high, or a single dose. All individual treatments were compared to the CTR using the contrast option in SAS. A total of 18 comparisons (8 treatments dosed at low and high doses, and 2 treatments dosed at single doses) were contrasted with the CTR. Results are reported as least squares means (±SEM), and unless otherwise specified, treatment effects were declared significant at *p* < 0.05.

## 3. Results

### 3.1. Rumen Fermentation

Treatments had a significant overall effect on total VFA (TVFA) concentrations (*p* < 0.01). ORE_High, GAR_High, GAR_Low and CIN_High showed significantly lower TVFA concentrations compared with the CTR, whereas none of the remaining EO treatments differed significantly from the CTR.

Overall treatment effects on the major VFA proportions were significant (*p* < 0.01). GAR caused a marked metabolic shift (*p* < 0.01), at both doses (GAR_High and GAR_Low) it resulted in the most substantial reductions in acetate molar proportion (*p* < 0.01), concurrent with the most substantial increases in propionate molar proportion. These coordinated shifts also resulted in the most marked reductions in the A:P ratio. MON also increased propionate molar proportion and reduced the A:P ratio. In contrast, ORE_High and LEG_High resulted in the greatest reductions in propionate molar proportion along with significant increases in the A:P ratio.

Butyrate molar proportion varied significantly among treatments (*p* < 0.01), with ORE_High, GAR_High, GAR_Low, and LEG_High exhibiting significantly higher butyrate proportions compared with the CTR, whereas MON_SNGL showed a significant reduction. Valerate proportion likewise differed among treatments (*p* < 0.01); the highest values were observed for LEG_High, GAR_Low, and GAR_High, while CIN_Low exhibited a significant reduction.

Branched-chain VFA (BCVFA) molar proportions were significantly affected by treatments (*p* < 0.01). The highest values were recorded for PPM_High, LEG_High, and LAV_High, while the lowest value was observed with ORE_High. NH_3_-N concentration also differed significantly among treatments (*p* < 0.01). The most significant reductions were observed with ORE_High, MON_SNGL, and CIN_High, whereas COR_High showed a marked increase relative to the CTR.

The initial pH was adjusted to 6.9, and after 24 h of incubation, a significant decrease was observed (*p* < 0.01). Most treatments resulted in higher pH values after 24 h than the CTR (6.08); however, CIN at a low dose produced a significantly lower pH value (5.93).

To facilitate interpretation of treatment effects, the results at 24 h post-incubation are presented in the main table (Table 2), as this time point provides the most informative indication of cumulative fermentation. The corresponding 6 h values are provided in Supplementary Table S1 to document early-stage fermentation responses. This approach highlights the principal effects of additive type and dose while maintaining clarity and interpretability.

### 3.2. Gas and CH_4_ Production

**Figure 1:**
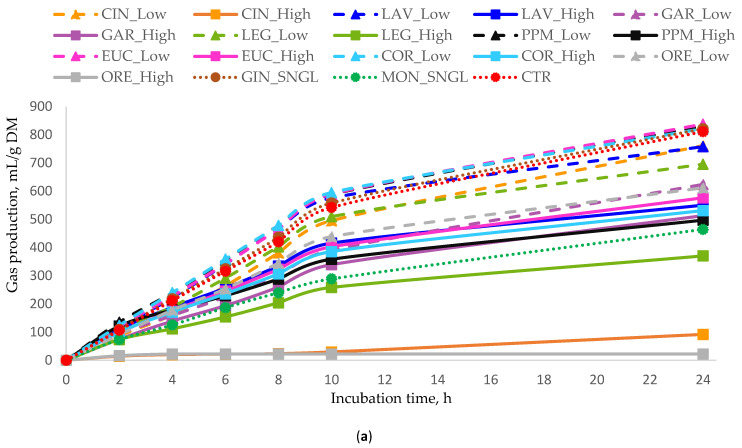

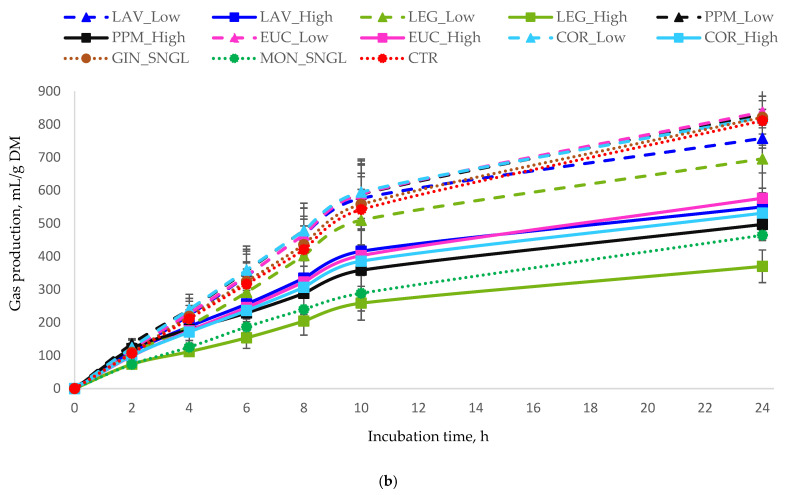

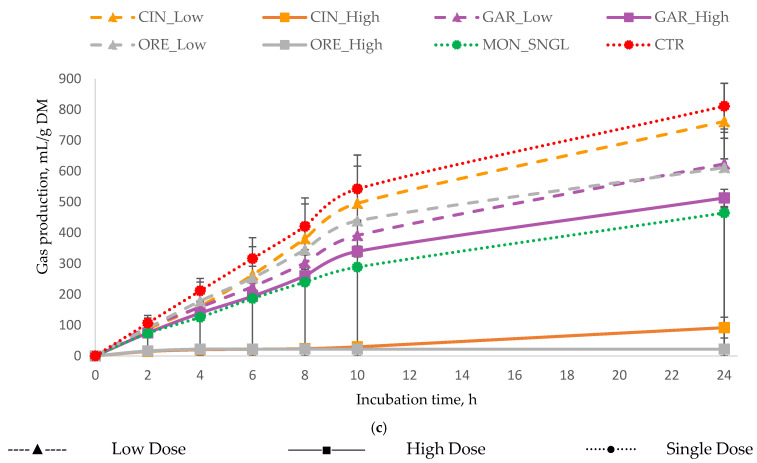
In vitro cumulative gas production (mL of gas/g DM) as a function of incubation time for (**a**) all essential oils, (**b**) terpenoid essential oils, and (**c**) non-terpenoid essential oils. Monensin (MON) and the control (CTR) are included in all panels as reference treatments. For each treatment, inclusion levels are distinguished by line style and marker type. Error bars represent standard deviation (SD) and are shown only in grouped panels to illustrate variability while maintaining figure clarity. Treatments: LAV = lavender; LEG = lemongrass; PPM = peppermint; EUC = eucalyptus; COR = coriander; GIN = ginger; CIN = cinnamon; ORE = oregano; GAR = garlic; MON = monensin; CTR = control.

**Figure 2:**
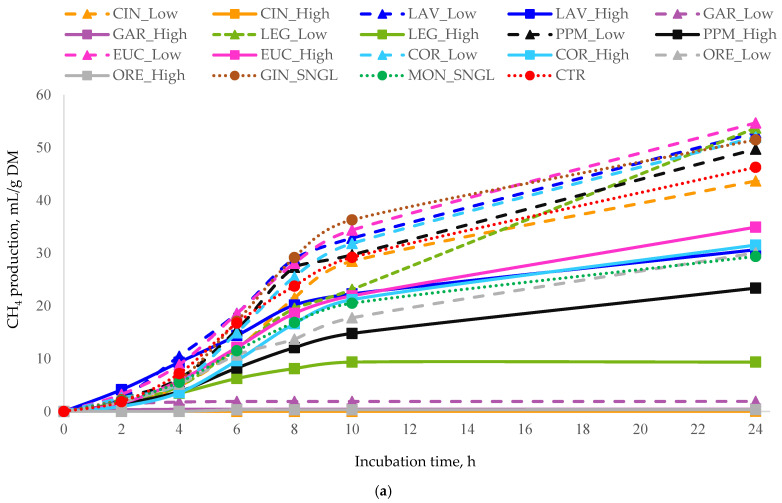

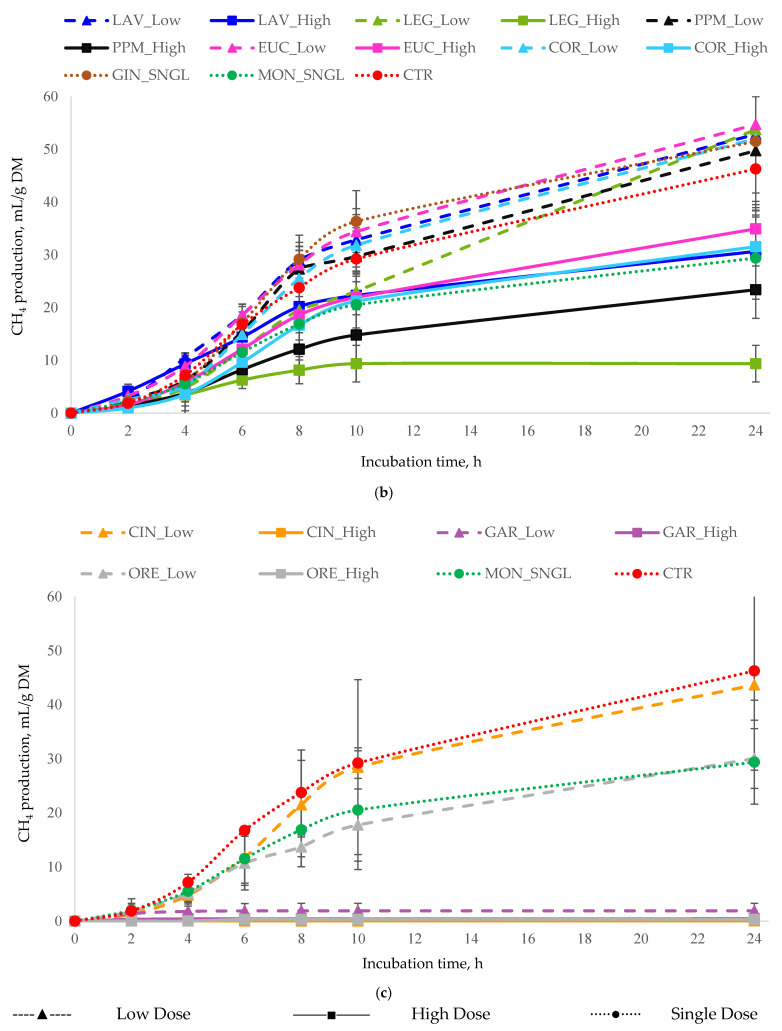
In vitro cumulative CH_4_ production (mL of CH_4_/g DM) as a function of incubation time for (**a**) all essential oils, (**b**) terpenoid essential oils, and (**c**) non-terpenoid essential oils. Monensin (MON) and the control (CTR) are included in all panels as reference treatments. For each treatment, inclusion levels are distinguished by line style and marker type. Error bars represent standard deviation (SD) and are shown only in grouped panels to illustrate variability while maintaining figure clarity. Treatments: LAV = lavender; LEG = lemongrass; PPM = peppermint; EUC = eucalyptus; COR = coriander; GIN = ginger; CIN = cinnamon; ORE = oregano; GAR = garlic; MON = monensin; CTR = control.

Among the treatments administered at different doses, the majority elicited significant reductions (*p* < 0.05) in cumulative gas production, CH_4_ emission, and the CH_4_/total gas ratio when compared to the CTR. The cumulative total gas and CH_4_ production values at 24 h are presented in Table 3, while their temporal production profiles are illustrated in Figure 1 and Figure 2, respectively.

Substantial variation was observed in the reduction of cumulative gas production at 24 h among treatments, with decreases ranging from −14% to −54% (from 696 to 370 gas mL/g DM), compared to the CTR (Table 3). The greatest reduction in total gas production was observed with treatment LEG_High, followed by MON_SNGL, while PPM_High and GAR_High also resulted in substantial reductions. Some of the treatments showed a moderate decrease ranging from −23% to −35%. In contrast, none of the treatments increased total gas production compared with the CTR.

Patterns of CH_4_ production (Table 3) closely paralleled those observed for total gas production. GAR_High produced the lowest CH_4_ output and the lowest CH_4_/total gas ratio (consistent with its strong effect on total gas production), with GAR_Low and LEG_High showing the next lowest values.

In contrast, EUC_Low significantly increased CH_4_ production. Elevated CH_4_/total gas ratios were recorded for LEG_Low, followed by LAV_Low and EUC_Low. Overall, gas and CH_4_ production responses were treatment and dose-dependent, with high-dose treatments demonstrating the greatest efficacy compared to low-dose or single-dose treatments.

## 4. Discussion

An ideal feed additive for ruminants is one that simultaneously suppresses CH_4_, improves fermentation efficiency, and enhances nitrogen utilization. In practical terms, desirable outcomes include maintaining or increasing TVFA concentrations, shifting VFA profiles toward greater propionate and a lower A:P ratio, reducing NH_3_-N levels, and limiting CH_4_ emissions [25]. However, optimal rumen function depends on the coordinated regulation of multiple parameters [46], and focusing exclusively on one parameter risks driving the system toward suboptimal fermentation efficiency [47]. Hence, the value of an “effective” rumen modifier lies in its holistic balance of fermentation effects, rather than any single outcome. In this context, the present study examines the impact of individual EOs on rumen fermentation and CH_4_ emissions, focusing on two categories: terpenoid EOs and non-terpenoid EOs. The overall treatment effect was significant (*p* < 0.01) on key fermentation parameters, including TVFA, VFA molar proportions, NH_3_-N, and pH, suggesting the high responsiveness of rumen fermentation to supplementation with these bioactive compounds. These findings are consistent with previous studies demonstrating that plant-derived bioactives can modulate microbial activity and fermentation end-products [48,49].

Essential oils exert their effects in the rumen through diverse biochemical mechanisms [16], which are strongly influenced by their dominant chemical constituents [50]. In this study, nine EOs were tested, classified as terpenoid (LAV, LEG, PPM, EUC, COR, GIN) and non-terpenoid EOs (GAR, ORE, CIN), allowing interpretation of their contrasting effects on fermentation and CH_4_.

### 4.1. Terpenoid EOs

Six oils (LAV, LEG, PPM, EUC, COR, GIN) belonged to the terpenoid group, consisting primarily of monoterpenes and one sesquiterpene. In the present work, they produced dose-dependent effects with overall modest CH_4_ reductions and largely stable fermentation, with the exception of specific shifts in VFA profiles. Unlike the Non-Terpenoid EOs, their activity did not depress total fermentation intensity (TVFA), although certain compounds (e.g., LEG, PPM, LAV) modified VFA distribution at effective doses. This pattern reflects the comparatively weaker antimicrobial pressure of terpenoids, attributed to the absence of a phenolic ring and highly polar substituents responsible for strong, non-selective membrane disruption [51]. Instead, their effects stem from lipophilic membrane interactions that alter permeability and microbial metabolism more selectively, leading to targeted or subtle shifts rather than broad inhibition [52].

Lemongrass oil (LEG), dominated by citral, displayed a clear antimicrobial threshold. At the lower dose, fermentation remained largely unchanged, and CH_4_ formation was unaffected, indicating insufficient biochemical pressure to disrupt methanogenesis, consistent with earlier reports of limited activity at sub-effective citral levels [37,53]. In contrast, the higher dose strongly suppressed CH_4_ production, in agreement with citral-based inhibition reported by Joch et al. [36] and Wanapat et al. [54]. This response reflects citral’s capacity to disrupt microbial membranes and interfere with intracellular processes [55]. However, although TVFA did not decline significantly, the marked reduction in total gas production (−54%) indicates broad inhibition of fermentative activity rather than selective targeting of methanogenesis. Fermentation balance was also altered, as propionate declined and butyrate increased, indicating that the antimicrobial pressure exceeded a selective range and impaired H_2_-utilizing propionate producers. This contrasts with Temmar et al. [28], where low citral doses enhanced propionate formation, whereas the higher concentration used here shifted the rumen toward a less favorable VFA profile. Therefore, although LEG can reduce CH_4_, its efficacy is compromised by unfavourable redirection of fermentation pathways at high doses, making such application unsuitable for efficient rumen functioning.

In contrast, PPM, enriched in menthol, produced a more desirable mitigation profile. At low inclusion, CH_4_, total gas, and VFA distribution remained unchanged, in agreement with observations that sub-inhibitory menthol concentrations exert negligible rumen effects [33]. At the higher dose, however, PPM achieved substantial CH_4_ and acceptable gas reduction without decreasing TVFA, confirming effective suppression of methanogenesis without compromising fermentation capacity, consistent with findings of Özkan et al. [56] and Canbolat et al. [57]. Comparable selectivity has also been reported in buffalo rumen inoculum by Roy et al. [58], who observed strong CH_4_ inhibition across multiple PPM doses while fermentation remained largely unaffected, supporting the cross-species robustness of menthol-based mitigation. Menthol likely reduced CH_4_ through membrane disruption, targeting methanogens and interference with protozoa–methanogen associations [59]. Notably, only minor shifts were observed in secondary VFAs, i.e., valerate and BCVFA, suggesting limited diversion of reducing equivalents rather than metabolic impairment. Thus, PPM provided selective CH_4_ inhibition without inducing the broader fermentation trade-offs observed with high-dose LEG, indicating stronger application potential.

Lavender (LAV) and coriander (COR) exhibited nearly identical responses attributable to their shared linalool dominance. At low inclusion, neither affected CH_4_ nor fermentation, aligning with observations by Khan [60] who reported that linalool is largely inert below a functional threshold. At high inclusion, both reduced CH_4_ and total gas while preserving TVFA, indicating CH_4_ suppression without compromising fermentative output, consistent with an in vitro study by Roy et al. [58]. Linalool likely acts by limiting H_2_ supply through antimicrobial action on fibrolytic and saccharolytic communities [61], resulting in indirect CH_4_ reduction. Only minor shifts in secondary VFAs (mainly valerate and BCVFA with LAV) were observed, reflecting small-scale diversion of remaining reductants [62]. Overall, the significant CH_4_ reduction observed with LAV and the comparable numerical trend with COR suggest a shared linalool-driven limitation of H_2_ supply that restricts CH_4_ formation while largely preserving primary rumen fermentation pathways.

Eucalyptus (EUC) and ginger (GIN) showed no measurable effects on CH_4,_ gas production, VFA profiles, or nitrogen indices, indicating a lack of antimicrobial potency at the applied levels. Given their neutral response, these treatments were not included in the detailed discussion.

With respect to nitrogen metabolism, only citral and linalool-based terpenoids elicited detectable responses. LEG decreased NH_3_-N at low dose, suggesting improved ammonia assimilation, but increased BCVFA without reducing NH_3_-N at high dose, indicative of intensified branched-chain amino acid (BCAA) deamination under antimicrobial stress [63]. COR, in contrast, increased NH_3_-N without altering BCVFA, consistent with impaired ammonia incorporation due to linalool’s disproportionate inhibition of NH_3_-assimilating microbes [64,65]. Thus, when effects occurred, terpenoids tended to reduce microbial nitrogen retention either by enhanced deamination (citral) or reduced NH_3_ assimilation (linalool), whereas menthol-rich and other terpenoids remained largely neutral.

In summary, terpenoid oils exhibited selective antimethanogenic potential that became evident only when their structural bioactive threshold was reached. At effective doses, LEG reduced CH_4_ but disrupted rumen fermentation balance, whereas PPM suppressed CH_4_ while preserving core VFA metabolism. Linalool-based extracts (LAV, COR) delivered moderate yet fermentation-stable CH_4_ reductions driven by limited H_2_ supply, and EUC and GIN showed no measurable bioactivity. These results emphasize that the mitigation value of terpenoids is determined less by dose alone and more by the chemical specificity of the active molecule in achieving targeted methanogen inhibition without compromising fermentative energetics.

### 4.2. Non-Terpenoid EOs

Non-terpenoid EOs such as ORE (carvacrol-rich), CIN (cinnamaldehyde-rich), and GAR (containing organosulfur constituents like DADS) are reported to exert comparatively strong antimicrobial effects on rumen microbiota [33,66,67]. Although these compounds differ chemically, carvacrol, being phenolic, cinnamaldehyde, an aromatic phenylpropanoid, and garlic constituents, organosulfur, they display functionally convergent modes of action involving membrane disruption and interference with microbial enzymatic processes [68]. In this study, these non-terpenoid EOs exhibited a shared tendency to depress TVFA production and alter fermentation patterns, particularly at higher inclusion levels. At lower doses, their effects diverged, with responses that were generally comparable to the CTR and only modest reductions in gas and CH_4_. Among these, GAR distinguished itself by achieving strong CH_4_ mitigation while maintaining a favorable fermentation pattern across both inclusion levels, making it the most promising candidate EO under the conditions tested.

Garlic oil (GAR) showed the highest antimethanogenic efficacy, suppressing CH_4_ by >90% at both inclusion levels while maintaining a favorable fermentation pattern despite moderate decrease in fermentation intensity. These responses are strongly supported by previous in vitro studies using comparable concentrations of organosulfur compounds. Busquet et al. [69] reported substantial CH_4_ inhibition together with reduced TVFA at 300 mg/L, closely aligning with the combined profile of strong CH_4_ suppression and moderated fermentation intensity observed in the present study. Likewise, Dey et al. [70] documented dose-dependent CH_4_ reductions (approximately 40–75%) in buffalo rumen fluid at doses up to 153 mg/L, consistent with the selectivity exhibited by our lower GAR dose and its redirection of reducing equivalents toward more energetically favorable pathways. This convergence of findings across species and experimental systems reinforces the robustness of the response observed for GAR in this study. The magnitude of inhibition is also consistent with the high diallyl sulfide activity described by Soliva et al. [71], reinforcing that strong CH_4_ abatement can be achieved without broad fermentation collapse. Unlike the generalized metabolic suppression reported for certain phenolic compounds [72], GAR acted by targeting methanogens and protozoa [73,74] while sparing propionate-forming communities [32,33]. In contrast, results such as those of Nanon et al. [35], who reported increased CH_4_ and elevated TVFA at ≥200 mg/L, suggest that GAR’s selectivity may be dose-dependent and susceptible to loss when concentrations exceed an effective threshold. Collectively, these results indicate that GAR can provide potent and selective CH_4_ mitigation within an appropriate dosing range. Nevertheless, because the present responses were obtained under a 24 h in vitro batch system, they should be regarded as encouraging but preliminary, requiring validation in longer-term continuous-culture and in vivo evaluations.

Oregano oil (ORE) displayed a dose-sensitive response, selectively suppressing CH_4_ at low doses but impairing fermentation at high doses. At the lower inclusion level, CH_4_ decreased without affecting TVFA or VFA stoichiometry, consistent with controlled antimicrobial action seen at moderate carvacrol concentrations [75]. This activity is largely attributed to carvacrol’s hydroxylated aromatic structure, which promotes membrane disruption and inhibition of key microbial enzymes [68], allowing selective pressure on methanogenic pathways at lower doses. At higher inclusion levels, reductions in TVFA, total gas production, and propionate, along with an elevated A:P ratio, indicated non-specific inhibition of H_2_-dependent fermentation pathways rather than true metabolic redirection, similar to outcomes observed when carvacrol exceeds selective thresholds by Patra and Yu [33] and Zhou et al. [76]. Because propionate is a key H_2_ sink competing with methanogenesis [77], its suppression explains why additional CH_4_ reduction did not occur despite stronger antimicrobial pressure. For animal systems, ORE presents a very narrow effective window: low doses may offer CH_4_ mitigation without penalty, whereas higher levels represent false CH_4_ suppression driven by generalized fermentation inhibition and are unsuitable for practical dietary application.

Cinnamon oil (CIN) did not provide selective CH_4_ mitigation and compromised fermentation at high inclusion. At the lower dose, CIN showed no impact on CH_4_ or fermentation, consistent with the limited activity observed at sub-inhibitory cinnamaldehyde levels, as reported by Mateos et al. [78]. At high inclusion, reductions in CH_4_ coincided with sharp declines in gas and TVFA, indicating non-selective metabolic inhibition rather than targeted antimethanogenesis, a response similar to fermentation collapse reported at elevated cinnamaldehyde doses by Blanch et al. [79]. The concurrent reduction in propionate and rise in A:P ratio are consistent with cinnamaldehyde-mediated suppression of both fibrolytic and propionate-forming bacteria [67]. Because propionate suppression eliminates true H_2_ redirection, CH_4_ reduction under CIN reflects false mitigation. Thus, unlike GAR, which enhanced H_2_ productivity, CIN represents a metabolically counterproductive strategy and does not appear suitable for practical dietary use.

The functional divergence among GAR, ORE, and CIN seen in methanogenesis was mirrored in their differing effects on rumen nitrogen metabolism. ORE reduced both NH_3_-N and BCVFA at high inclusion, indicating broad suppression of proteolysis rather than improved nitrogen retention, consistent with excessive carvacrol pressure as reported by Zhou et al. [80] and Benchaar and Hassanat [81]. CIN reduced NH_3_-N without affecting BCVFA, suggesting selective inhibition of deaminating bacteria and restrained hyper-ammonia activity [38], whereas GAR remained neutral, indicating that its strong antimethanogenic action did not interfere with microbial protein pathways.

Taken together, non-Terpenoid EOs differed as much in mechanism as in efficacy. GAR achieved selective CH_4_ mitigation with favorable H_2_ redirection; ORE provided dose-dependent benefits, with the high inclusion level showing an apparent CH_4_ reduction driven by overall fermentation suppression; CIN caused non-selective fermentation inhibition and lacked practical value. These divergent outcomes highlight that effective rumen modification depends not only on bioactive potency but also on chemical structure, bioactive targets, and dose precision.

Clear patterns emerged when comparing responses across and within the two EO classes. The terpenoid oils produced modest, dose-dependent CH_4_ reductions while largely preserving overall fermentation intensity, with effects expressed mainly through targeted shifts in VFA proportions. Within this group, PPM and LEG provided a particularly informative contrast: both suppressed CH_4_ at their higher doses, but PPM did so while maintaining TVFA and only minimally altering VFA profiles, whereas LEG at higher doses coupled strong CH_4_ suppression with a marked reduction in total gas production and a shift toward a less favorable VFA pattern. This divergence likely reflects chemical-structural differences—menthol exerting more selective antimicrobial pressure, while citral more readily spills over into non-specific inhibition. The linalool-rich oil LAV showed a more moderate response profile particularily at lower doses, producing moderate CH_4_ reductions accompanied by a stable fermentation pattern, consistent with the comparatively mild, membrane-modulating activity of linalool-based compounds. In contrast, the non-terpenoid oils displayed stronger and less selective antimicrobial activity. Among them, ORE and CIN reduced CH_4_ only at doses that also depressed TVFA and gas production and disrupted propionate formation, indicating dose-limited selectivity typical of phenolic and aromatic compounds. GAR, however, differed markedly from the other non-terpenoids: despite strong CH_4_ suppression at both doses, it maintained a favorable VFA pattern and caused only moderate reductions in fermentation intensity. This selective profile is consistent with the targeted activity of organosulfur compounds against methanogens and protozoa, in contrast to the broader inhibition observed for carvacrol and cinnamaldehyde. Together, these patterns show that both the magnitude and selectivity of CH_4_ mitigation depend not only on dose but fundamentally on EO chemical architecture and the microbial functions it targets.

Overall, the EOs demonstrated variable but meaningful potential for CH_4_ mitigation, with effects that were both dose-dependent and strongly shaped by chemical structure. Several EO treatments achieved substantial reductions in CH_4_ while maintaining stable fermentation profiles. Among the non-terpenoid EOs, GAR showed the strongest and most consistent antimethanogenic response, reduced CH_4_ by more than 90% across doses while inducing only moderate decreases in fermentation intensity alongside a favorable restructuring of rumen fermentation patterns. Within the terpenoid group, PPM provided the most balanced outcome, achieving a significant CH_4_ inhibition at the higher dose without compromising overall fermentation balance. By contrast, LEG also reduced CH_4_ but exhibited clearer fermentation depression when supplemented at a higher dose. As expected for compounds with broad antimicrobial activity, higher doses of several EOs suppressed total fermentation even when CH_4_ inhibition remained evident. These findings underscore that while multiple EOs can contribute to CH_4_ mitigation, their suitability as feed additives ultimately depends on achieving selective action at appropriate doses, reinforcing the need for continued systematic screening and mechanism-based evaluation to support the development of effective, sustainable enteric CH_4_ reduction strategies.

This study adopted a broad in vitro screening strategy to evaluate a diverse set of essential oils, with terpenoid EOs comprising most treatments and a smaller set of structurally distinct non-terpenoid EOs included for comparison. While this design enabled meaningful assessment of relative efficacy, the unequal representation of EO classes and the use of only two inclusion levels limit deeper class-specific interpretation and restrict the definition of dose–response patterns. These constraints are inherent to the exploratory nature of the work and indicate clear priorities for future research, including balanced class representation, expanded dosing ranges and targeted combinations of complementary bioactives for CH_4_ mitigation and improved rumen fermentation. Overall, the findings demonstrate that EOs differ markedly in their capacity and selectivity to mitigate CH_4_, reinforcing the need for systematic screening supported by mechanistic insight to inform the development of effective and sustainable feed-additive strategies for enteric CH_4_ reduction.

## 5. Conclusions

This study demonstrates that EOs can markedly influence rumen fermentation and CH_4_ emissions, with effectiveness governed primarily by chemical structure and dose. The tested EOs exhibited responses ranging from selective CH_4_ suppression to broad inhibition of fermentative activity. Among them, GAR emerged as the most promising additive under the tested in vitro conditions, achieving consistent near-complete CH_4_ suppression across the tested doses while maintaining a favorable fermentation pattern, characterized by reduced acetate, increased propionate, and a lower A:P ratio. Although GAR also induced a moderate reduction in overall fermentation intensity, it preserved key fermentative pathways and sustained a balanced VFA profile, indicating a selective rather than nonspecific antimicrobial action. It should nevertheless be emphasized that this extent of CH_4_ inhibition reflects behavior observed within a 24 h in vitro batch culture conditions and should be interpreted within this context, as continuous-culture or in vivo systems may respond differently and require further verification. Peppermint oil (PPM) also reduced CH_4_ at its higher inclusion level without compromising core fermentation, indicating a selective and well-tolerated antimethanogenic effect.

Overall, these findings underscore that effective rumen CH_4_ mitigation requires bioactive compounds that redirect H_2_ metabolism toward productive pathways rather than simply enhance fermentation. Mechanism-driven additive selection and careful dose calibration are therefore critical to achieve this balance. Future work should evaluate synergistic combinations, characterize long-term microbial adaptation, and validate these mechanisms in vivo to support practical implementation in sustainable ruminant production systems.

## Figures and Tables

**Table 1:** Description of the different bioactive compounds used as treatments and their respective doses.

Treatments	Main Active Components ^2^	Dose (mg/L) ^3^	
		Low	High
Cinnamon EO, CIN	75% cinnamaldehyde	500	1200
Lavender EO, LAV	25% linalyle acetate, 29.9% linalol	500	1200
Garlic EO, GAR	50% DADS ^1^	150	350
Lemongrass EO, LEG	75% citral (néral + géranial)	500	1200
Peppermint EO, PPM	50% menthol	300	750
Eucalyptus EO, EUC	80% cineole	500	1200
Coriander EO, COR	70% linalool	300	750
Origano EO, ORE	70% carvacrol	300	750
Ginger EO, GIN	55% sesquiterpenes	150	
Monensin, MON	91% Monensin	12.5	

^1^ DADS = diallyl disulfide; ^2^ Information provided by TECHNA France (except for Monensin provided by Sigma-Aldrich); ^3^ Dose of the original product; Essential oil (EO).

**Table 2:** Effects of essential oils on in vitro rumen microbial fermentation parameters at 24 h post-incubation. Parameters include total volatile fatty acids (TVFAs), molar proportions, and ammonia-N (NH_3_-N) concentration.

Treatment ^1^	Fermentation Response Variables ^2^																
	TVFA		Molar Proportions, %										C2:C3 ^3^		NH_3_-N		Final pH
	(mM)		C2		C3		C4		Valerate		BCVFA		Ratio		(mg/dL)		
Terpenoid EOs																	
LAV_Low	150.2		70.2		12.6		12.5		1.41		3.40		5.56		34.8		6.00
LAV_High	149.6		69.5		11.9		13.1		1.60 *	+12.8	3.81 *	+9.70	5.78		35.5		6.16
LEG_Low	153.6		69.7		12.6		12.7		1.46		3.44		5.49		33.5 *	−6.10	6.09
LEG_High	139.0		68.8		10.1 *	−17.4	15.6 *	+27.1	1.75 *	+23.5	3.95 *	+13.9	6.93 *	+20.9	36.2		6.26 *
PPM_Low	146.9		70.5		12.1		12.5		1.43		3.57		5.92		35.5		6.13
PPM_High	138.5		68.3 *	−3.40	11.8		14.2 *	+16.1	1.65 *	+16.9	4.10 *	+18.2	5.91		35.1		6.30 *
EUC_Low	151.8		70.5		12.4		12.5		1.41		3.39		5.79		34.7		6.05
EUC_High	143.7		68.9		12.2		13.7		1.55		3.78 *	+8.90	5.85		35.1		6.18
COR_Low	151.9		70.2		12.4		12.5		1.40		3.42		5.61		35.8		6.00
COR_High	144.9		69.7		12.4		12.7		1.47		3.62		5.61		37.5 *	+5.10	6.12
GIN_SNGL	151.3		70.4		12.3		12.4		1.40		3.42		5.61		34.8		6.07
Non-terpenoid EOs																	
CIN_Low	153.5		70.7		11.8		13.0		1.35 *	−4.80	3.16		5.89		35.4		5.93 *
CIN_High	132.7 *	−11.4	68.2 *	−3.54	11.3 *	−7.20	15.1 *	+23.4	1.67 *	+18.3	3.76		6.03 *	+5.30	33.4 *	−6.60	6.24 *
ORE_Low	143.7		69.6		12.4		13.0		1.44		3.55		5.72		35.5		6.21 *
ORE_High	105.7 *	−29.5	69.1		9.77 *	−19.8	16.4 *	+34.3	1.37		3.08 *	−11.4	6.96 *	+21.5	30.1 *	−15.7	6.5 *
GAR_Low	129.6 *	−13.5	62.1 *	−12.1	16.8 *	+38.1	15.9 *	+30.3	1.73 *	+22.2	3.36		3.76 *	−34.4	35.5		6.24 *
GAR_High	126.9 *	−15.3	61.9 *	−12.5	16.9 *	+38.6	16.1 *	+31.2	1.71 *	+21.2	3.35		3.67 *	−35.9	35.3		6.29 *
Reference Compounds																	
MON_SNGL	137.2 *	−8.50	68.5 *	−3.10	15.1 *	+24.3	11.5 *	−5.90	1.45		3.75		4.59 *	−19.9	32.5 *	−8.90	6.32 *
CTR	149.9		70.7		12.2		12.2		1.42		3.47		5.73		35.7		6.08
SEM ^4^	3.94		1.04		0.68		1.27		0.046		0.23		0.27		0.81		0.045
*p*-Value	<0.01		<0.01		<0.01		<0.01		<0.01		<0.01		<0.01		<0.01		<0.01

^1^ Treatment = Tested additives in either single or dual (Low and High) doses. LAV = lavender; LEG = lemongrass; PPM = peppermint; EUC = eucalyptus; COR = coriander; GIN = ginger; CIN = cinnamon; ORE = oregano; GAR = garlic; MON = monensin; CTR = control. All treatments are essential oils except MON and CTR. Values to the right of columns are color-coded and represent the percentage change (either increasing or decreasing) compared to the CTR (* *p* < 0.05). Values in ‘green’ indicate a desirable effect and ‘red’ an undesirable effect. ^2^ VFA = Volatile Fatty Acids; C2 = Acetate; C3 = Propionate; C4 = Butyrate; BCVFA = Branched Chain VFA. ^3^ Acetate-to-propionate ratio. ^4^ SEM = Standard Error of the Mean; *p*-Value of the fixed effects of treatment.

**Table 3:** Total gas, methane (CH_4_) production, CH_4_ to total gas ratio, and pH after 24 h of in vitro fermentation.

Treatments ^1^	Gas and Methane Production					
	Cumulative Total Gas (mL/g DM)		Cumulative CH_4_ (mL CH_4_/g DM)		Ratio CH_4_/Total Gas (%)	
Terpenoid EOs						
LAV_Low	758		52.8		6.99 *	+19
LAV_High	550 *	−32	30.6 *	−34	5.56	
LEG_Low	696 *	−14	53.7		8.33 *	+41
LEG_High	370 *	−54	9.37 *	−80	2.50 *	−58
PPM_Low	831		49.7		6.04	
PPM_High	497 *	−39	23.4 *	−50	4.67	
EUC_Low	838		54.7 *	+18	6.60 *	+12
EUC_High	576 *	−29	34.9		6.06	
COR_Low	822		52.0		6.36	
COR_High	531 *	−35	31.5		5.99	
GIN_SNGL	822		51.5		6.23	
Non-terpenoid Eos						
CIN_Low	761		43.7		5.76	
CIN_High	-		-		-	
ORE_Low	612 *	−25	30.1 *	−35	4.97	
ORE_High	-		-		-	
GAR_Low	624 *	−23	1.91 *	−96	0.31 *	−95
GAR_High	513 *	−37	0.44 *	−99	0.09 *	−98
Reference Compounds						
MON_SNGL	464 *	−43	29.4 *	−37	6.32	
CTR	811		46.3		5.90	
SEM ^2^	28.2		5.70		0.88	
*p*-Value	<0.01		<0.0001		<0.0001	

^1^ Treatment = Tested additives in either single or dual (Low and High) doses. LAV = lavender; LEG = lemongrass; PPM = peppermint; EUC = eucalyptus; COR = coriander; GIN = ginger; CIN = cinnamon; ORE = oregano; GAR = garlic; MON = monensin; CTR = control. All treatments are essential oils except MON and CTR. Values to the right represent the percentage change (either increasing or decreasing) compared to the CTR (* *p* < 0.05). Values are color-coded: ‘green’ indicate a desirable effect and ‘red’ an undesirable effect. ^2^ SEM = Standard Error of the Mean.

## Data Availability

The data presented in this study are available upon request from the corresponding author.

## Supplementary Materials

The following supporting information can be downloaded at https://www.mdpi.com/article/10.3390/ani16030373/s1. Supplementary Table S1: Effects of essential oils on in vitro rumen fermentation parameters at 6 h post-incubation.

## Abbreviations

The following abbreviations are used in this manuscript:

A:P	Acetate-to-propionate ratio
BCVFA	Branched-chain VFA
CH_4_	Methane
EOs	Essential oils
H_2_	Hydrogen
NH_3_-N	Ammonia-N
TVFA	Total VFAs
VFA	Volatile fatty acid
