# Supplementary material for: In Vitro Assessment of Essential Oils for Their Methane Mitigation Potential and Impact on Rumen Fermentation in Cattle
Source: Animals (Basel). 2026 Jan 24;16(3):373. doi: 10.3390/ani16030373 (PMC12897239; doi:10.3390/ani16030373)
Supplement: Supplementary file 1 [file animals-16-00373-s001.zip › animals-4101504-supplementary.pdf]

Supplementary Table S1: Effects of essential oils on *in vitro* rumen microbial fermentation parameters at 6 h post-incubation. Parameters include total volatile fatty acids (TVFAs), molar proportions, and ammonia-N (NH3-N) concentration.

Treatment[1]	Fermentation response variables[2]															
	TVFA (mM)		Molar proportions, %										C2:C3[3] Ratio		NH_3-N (mg/dL)	
			C2		C3		C4		Valerate		BCVFA					
Terpenoid EOs																
LAV_Low	103.9		70.8		13.5		11.3		1.34		3.32		5.25		31.0	
LAV_High	105.4		69.8		13.0		11.9		1.48*	+8.75	3.61*	+6.64	5.25		33.6*	+5.20
LEG_Low	100.1		69.5		13.5		12.2*	+8.60	1.46		3.60		5.16		33.0	
LEG_High	101.9		70.7		12.0*	-11.8	12.7*	+12.9	1.50*	+10.3	3.23		6.02*	+17.9	33.1*	+3.80
PPM_Low	106.0		70.3		13.4		11.4		1.35		3.39		5.32		29.4	
PPM_High	102.8		69.1*	-1.58	13.5		11.9		1.45		3.65*	+7.87	5.07		31.1	
EUC_Low	104.5		70.8		13.4		11.2		1.36		3.39		5.29		29.1	
EUC_High	106.3		70.1		13.4		11.5		1.43		3.48		5.32		30.6	
COR_Low	105.0		70.5		13.4		11.3		1.36		3.38		5.29		28.4*	-10.9
COR_High	98.3		68.7*	-2.23	13.7		11.9		1.50*	+9.90	3.82*	+13.0	5.00		31.4	
GIN_SNGL	100.9		70.4		13.6		11.3		1.37		3.47		5.26		30.4	
Non-terpenoid EOs																
CIN_Low	95.7		70.0		13.2		12.1		1.45		3.45		5.36		29.9	
CIN_High	90.0*	-11.7	70.1		12.6*	-6.70	12.6*	+12.1	1.50*	+9.90	3.45		5.73*	+12.1	30.1	
ORE_Low	101.5		69.9		13.6		11.4		1.40		3.55		5.07		29.8	
ORE_High	90.3*	-11.4	70.9		11.9*	-12.2	12.9*	+15.3	1.28*	-6.10	3.38		6.11*	+19.6	26.4*	-17.3
GAR_Low	93.6*	-8.10	66.8*	-4.83	15.1*	+11.5	13.2*	+17.4	1.52*	+11.8	3.37		4.46*	-12.6	29.8	
GAR_High	93.0*	-8.70	66.7*	-5.00	15.1*	+11.7	13.2*	+18.0	1.52*	+11.6	3.28		4.43*	-13.3	30.2	
Reference Compounds																
MON_SNGL	97.7		69.1*	-1.59	14.2*	+5.05	11.7		1.31*	-3.93	3.48		4.82*	-5.59	32.1	
CTR	101.9		70.2		13.6		11.2		1.36		3.39		5.11		31.9	
SEM[4]	3.93		0.57		0.58		1.08		0.13		0.091		0.22		2.08	
P-Value	<0.01		<0.01		<0.01		<0.01		<0.01		<0.01		<0.01		<0.01	

[1] Treatment = Tested additives in either single or dual (Low and High) doses. LAV = lavender; LEG = lemongrass; PPM = peppermint; EUC = eucalyptus; COR = coriander; GIN = ginger; CIN = cinnamon; ORE = oregano; GAR = garlic; MON = monensin; CTR = control. All treatments are essential oils except MON and CTR. Values to the right of columns are color-coded and represent the percentage change (either increasing or decreasing) compared to the CTR (* $P < 0.05$). Values in 'green' indicate a desirable effect and 'red' an undesirable effect. [2] VFA= Volatile Fatty Acids; C2= Acetate; C3= Propionate; C4= Butyrate; BCVFA = Branched Chain VFA. [3] Acetate to propionate ratio. [4] SEM = Standard Error of the Mean; P-Value of the fixed effects of treatment.
